# Low-invasive somatic oncogenes and lymph node metastasis in pediatric papillary thyroid cancer: implications for prophylactic central neck dissection

**DOI:** 10.1530/ETJ-23-0265

**Published:** 2024-08-02

**Authors:** Julia A Baran, Mya Bojarsky, Stephen Halada, Julio C Ricarte-Filho, Amber Isaza, Aime T Franco, Lea F Surrey, Tricia Bhatti, Zubair Baloch, N Scott Adzick, Sogol Mostoufi-Moab, Ken Kazahaya, Andrew J Bauer

**Affiliations:** 1 Division of Endocrinology and Diabetes, The Thyroid Center, Children’s Hospital of Philadelphia, Philadelphia, Pennsylvania, USA; 2 Department of Pathology and Laboratory Medicine, Children’s Hospital of Philadelphia, University of Pennsylvania, Philadelphia, Pennsylvania, USA; 3 Department of Surgery, Children’s Hospital of Philadelphia, Philadelphia, Pennsylvania, USA; 4 Division of Oncology, Children’s Hospital of Philadelphia, Philadelphia, Pennsylvania, USA; 5 Division of Pediatric Otolaryngology, Children’s Hospital of Philadelphia, Philadelphia, Pennsylvania, USA; 6 Department of Otorhinolaryngology: Head and Neck Surgery, University of Pennsylvania, Philadelphia, Pennsylvania, USA

**Keywords:** pediatrics, somatic oncogenes, thyroid cancer

## Abstract

**Objective:**

The American Thyroid Association (ATA) Pediatric Guidelines recommend selective, prophylactic central neck dissection (pCND) for patients with papillary thyroid carcinoma (PTC) based on tumor focality, tumor size, and the surgeon’s experience. With the expansion of pre-surgical somatic oncogene testing and continued controversy over the benefits of pCND, oncogenic alteration data may provide an opportunity to stratify pCND. This study compared lymph node (LN) involvement in pediatric patients with PTC between tumors with low- and high-invasive-associated alterations to explore the potential utility of preoperative oncogenic alterations in the stratification of pCND.

**Methods:**

This is retrospective cohort study of pediatric patients who underwent somatic oncogene testing post thyroidectomy for PTC between July 2003 and July 2022.

**Results:**

Of 192 eligible PTC patients with postoperative somatic oncogene data, 19 tumors harbored somatic alterations associated with low-invasive disease (19/192, 10%), and 128 tumors harbored a *BRAF^V600E^* alteration (45/192, 23%) or an oncogenic fusion (83/192, 43%). Tumors with low-invasive alterations were less likely to present malignant preoperative cytology (2/18, 11%) than those with high-invasive alterations (97/124, 78%; *P* < 0.001). Twelve patients with low-invasive alterations had LNs dissected from the central neck (12/19, 63%) compared to 127 patients (127/128, 99%) with high-invasive alterations. LN metastasis was identified in two patients with low-invasive alterations (2/19, 11%) compared to 107 patients with high-invasive alterations (107/128, 84%; *P* < 0.001).

**Conclusion:**

Pediatric patients with low-invasive somatic oncogenic alterations are at low risk for metastasis to central neck LNs. Our findings suggest that preoperative knowledge of somatic oncogene alterations provides objective data to stratify pediatric patients who may not benefit from pCND.

## Graphical abstract


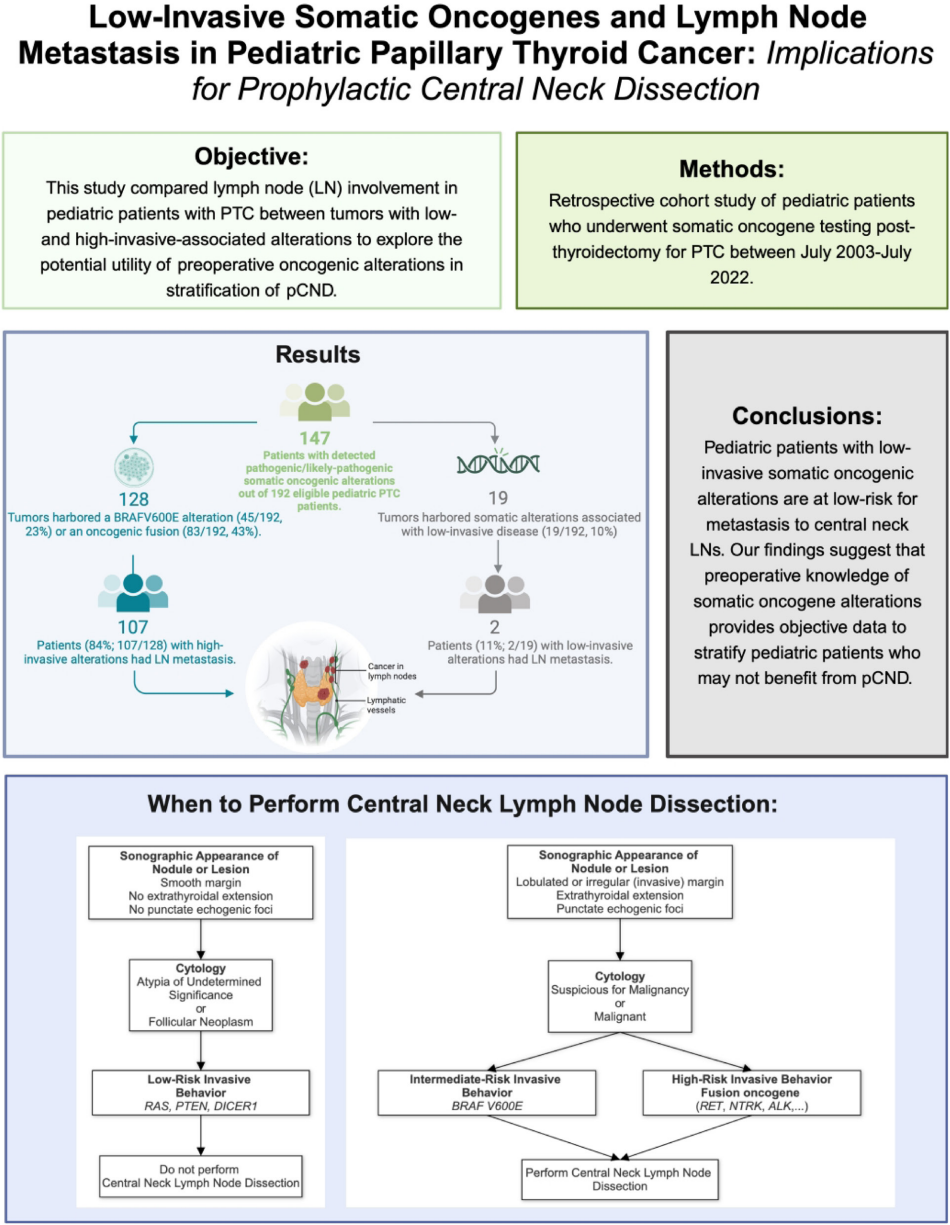


## Introduction

Papillary thyroid carcinoma (PTC) is the most common pediatric endocrine malignancy. While long-term disease-specific survival approaches 100% for children and adolescents (1, 2), recurrence following treatment may affect up to 30% of patients (3, 4), with cervical lymph node metastasis (LNM) in central and lateral neck compartments being well-established sites of persistent and recurrent disease. The 2015 American Thyroid Association (ATA) Pediatric Guidelines recommend selective consideration of prophylactic central neck dissection (pCND) for patients with PTC based upon tumor focality, tumor size, and the experience of the surgeon (Recommendation 12B) (5). This recommendation was based on data demonstrating a high rate of metastasis (30–90%) (6, 7) to regional lymph nodes (LNs) in the pediatric population and was used to stratify patients into ATA risk levels for selective use of radioiodine therapy (RAIT; Recommendation 15) (5, 8, 9). In addition, the extent of initial LN resection has been shown to impact disease free survival (DFS) (1, 5, 10). However, it is important to consider the potential benefit of pCND against the increased risk of surgical complications (11, 12). Based on this, the recent 2022 European Thyroid Association Guidelines for the management of pediatric thyroid nodules and differentiated thyroid carcinoma (DTC) recommended limiting pCND to patients with ‘suspicious features of advanced thyroid cancer’ (13).

Pre-operative assessment for surgical stratification commonly includes ultrasound (US) and fine needle aspiration (FNA), but these are highly subjective measures with wide variability in completeness and interpretation across institutions (14, 15, 16). Identifying a more objective preoperative marker that could predict the invasive behavior of the tumor, particularly tumors unlikely to metastasize to central neck LNs, would be clinically useful in decreasing reliance on pCND. If proven reliable, this marker would be associated with a high likelihood of surgical remission without placing patients at increased risk for potential surgical complications associated with pCND.

With the expansion of somatic oncogene analysis using next-generation sequencing (NGS), and data confirming associations between oncogenic alterations and invasive tumor behavior (17), knowledge of the somatic alteration preoperatively has the potential to further inform the surgical management of pediatric PTC (17, 18, 19, 20). Oncogene test results are not subject to variability in provider interpretation and offer impartial foresight into the pathological subtype and metastatic behavior of a patient’s disease (17, 21). This study was undertaken to compare LN involvement in pediatric patients with PTC demonstrating low- and high-invasive associated alterations to assess the association between molecular subtype and N1 disease in an effort to determine if somatic oncogene testing could be used to guide pCND stratification.

## Methods

### Data collection

An IRB-approved study was conducted on patients with PTC <19 years old who underwent thyroid surgery and somatic oncogene testing at the Children’s Hospital of Philadelphia (CHOP) between July 2003 and July 2022. Patient demographics, medical history, thyroid US, cytology, surgical approach, pathologic features, diagnosis, and postoperative somatic oncogene findings were extracted from the hospital electronic medical record system (Epic^®^). FNA cytology was classified according to The Bethesda System for Reporting Thyroid Cytopathology (TBSRTC) (22). A two-stage thyroidectomy was defined as a lobectomy followed by a completion thyroidectomy. Prophylactic CND (level VI or levels VI–VII) was performed for patients undergoing thyroidectomy without preoperative US evidence of central neck LN metastasis (American Joint Committee on Cancer (AJCC) N0a) and selected patients undergoing lobectomy with indeterminate (TBSRTC III–V) or malignant (TBSRTC VI) cytology. A therapeutic CND was performed for patients with preoperative evidence and FNA confirmation of central and lateral neck LN metastasis (AJCC N1b).

After surgery, primary tumor (T), regional LNs (N), and distant metastasis (M) were assigned using the 8th edition of the AJCC classification for PTC (23). Thyroid cancer risk stratification was adopted from the 2015 ATA Management Guidelines for Children with Thyroid Nodules and Differentiated Thyroid Carcinoma (5). Data analyses were limited to patients with a histological diagnosis of PTC.

### Molecular genetic analyses

Sequential somatic oncogene testing was performed on all tumors based on a histological diagnosis of PTC without selection based on AJCC TNM. Differing somatic panels were used over the length of the study based on availability and changes in clinical practice. Somatic alterations were identified using the following targeted assays: (i) CHOP’s Comprehensive Solid Tumor Panel (CSTP), (ii) Asuragen miRInform thyroid test, or (iii) Qiagen *BRAF* amplification test. CSTP comprises 238 genes and more than 600 fusions to detect single nucleotide variants, indels, gene fusions, and copy number alterations in pathologic specimens (24, 25). The Asuragen miRInform thyroid test analyzes the presence of common variants in *BRAF*,* HRAS*,* KRAS*, and *NRAS*, and fusion transcripts in *RET/PTC1*,* RET/PTC3*,and *PAX8-PPARγ* in pathologic specimens, and does not analyze *PTEN, DICER1*,* NTRK* fusions, and novel *RET* fusions (26). Qiagen detects somatic mutations in the *BRAF* oncogene utilizing RT-PCR (27).

### Statistical analysis

Genetic alterations were classified into two groups in accordance with previous studies demonstrating genotypic variations in behavior of pediatric PTC: low-invasive alterations (*DICER1*, *PTEN*, *RAS*, and *TSHR* variants, *BRAF* non*-V600E* variants; and *PAX8–PPARγ* fusion) and high-invasive alterations (*RET, NTRK, ALK, BRAF,* or* MET* fusions and *BRAF^V600E^* variant) (17, 18, 28, 29, 30). Patients with germline mutations in thyroid cancer predisposition syndromes, such as *PTEN* Hamartoma Tumor Syndrome and *DICER1* syndrome, were excluded from the analysis. Preoperative features, clinicopathologic characteristics, and outcomes were compared between patients with low- and high-invasive alterations. Categorical variables were summarized using frequency (percent) and were compared using the two-tailed Fisher exact test. Continuous variables were summarized using median (IQR = 1st–3rd quartiles) and were compared by Mann*–*Whitney *U* test (nonparametric). *P*-values ≤0.05 were considered statistically significant. All statistical analyses were performed in R 4.1.0 and R Studio 1.4.1717 with packages tidyverse and rstatix (31, 32, 33).

## Results

### Study cohort

A database of 192 pediatric PTC patients with postoperative somatic oncogene testing at CHOP was queried. Of the 192 eligible patients, 147 (147/192, 77%) patients had pathogenic/likely pathogenic (P/LP) somatic oncogenic alterations detected. Nineteen patients demonstrated P/LP somatic alterations associated with low-invasive disease (19/192, 10%), while 128 patients possessed somatic alterations associated with high-invasive disease (128/192, 67%), including *BRAF^V600E^* (45/192, 23%) and *RET*,* NTRK, ALK*,* BRAF*,or* MET* fusions (83/192, 43%; Table 1). The remaining 45 eligible patients were either found to not carry a somatic oncogenic alteration (40/192, 21%), harbor less frequent P/LP oncogenic alterations (3/192, 2%), including *RAD50*,* CHEK2*,or *BLM*, or carry germline variants (2/192, 1%). Of the 40 patients with no identifiable driver thyroid oncogenic alteration, 43% (17/40) underwent research molecular testing using the Asuragen miRinform Thyroid Test, which is not an NGS panel and does not test for fusion partners. These 17 samples were not available for retesting. The other 58% (23/40) underwent molecular testing using CSTP, but no variants/fusions were found.

**Table 1:** Demographics and medical history of pediatric patients with papillary thyroid carcinoma and somatic driver alterations confirmed by postoperative oncogene testing. Data are presented as *n* (%).

Characteristics	Values
Demographics	
Sex	
Male	38 (25.9)
Female	109 (74.1)
Race/ethnicity	
Asian	11 (7.5)
Black or African American	6 (4.1)
Hispanic or Latino	20 (13.6)
White	105 (71.4)
Other	9 (6.1)
Unknown/not reported	9 (6.1)
Medical history	
Radiotherapy for primary malignancy	
Yes	9 (6.1)
No	136 (92.5)
Unknown/not reported	2 (1.4)
Prior thyroid disease	
Yes	41 (27.9)
No	106 (72.1)
Family member with thyroid cancer	
Yes	21 (14.3)
No	122 (83.0)
Unknown/not reported	4 (2.7)
Next-generation sequencing panel	
Comprehensive solid tumor panel	123 (83.7)
Asuragen miRinform thyroid test	22 (15.0)
Qiagen amplification test	2 (1.4)
Driver alteration	
Low-invasive	
*BRAF nonV600E* variant^a^	2 (1.4)
*DICER1* variant	3 (2.0)
*PAX8–PPAR*γ fusion	2 (1.4)
*PTEN* variant	1 (0.7)
*RAS* variant	8 (5.4)
*TSHR* variant	3 (2.0)
High-invasive	
*ALK* fusion	8 (5.4)
*BRAF* fusion	5 (3.4)
*BRAF^V600E^* variant	45 (30.6)
*MET* fusion	4 (2.7)
*NTRK* fusion	24 (16.3)
*RET* fusion	42 (28.6)

^a^T599del and V600_K601delinsE.

### Preoperative, cytologic, and histologic features

**Figure 1:**
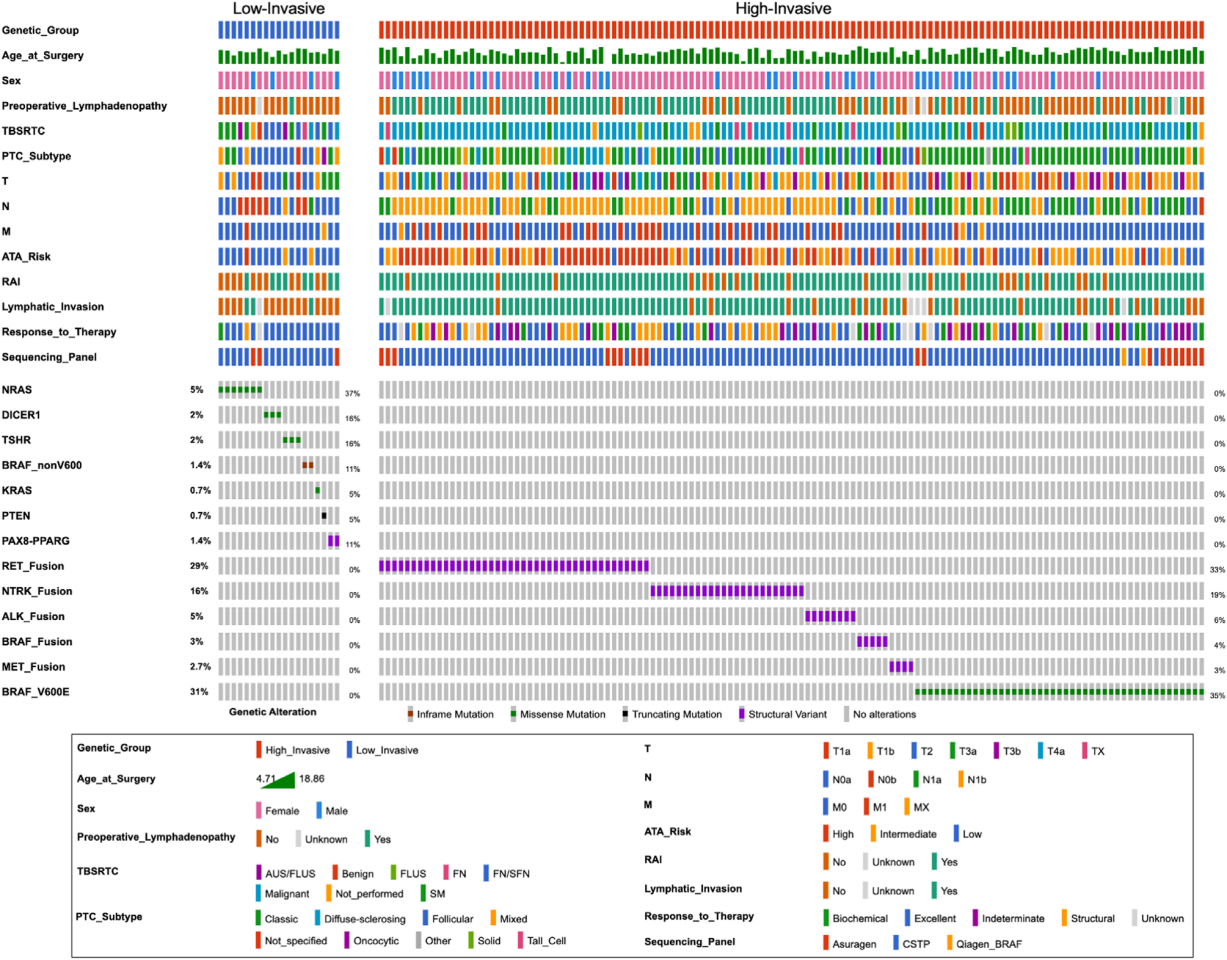
Clinicopathologic features of 19 patients with low-invasive and 128 patients with high-invasive somatic driver alterations who underwent thyroidectomy. Characteristics include age at the time of surgery, sex, preoperative lymphadenopathy, cytology (TBSRTC), histologic subtype, primary tumor (T) staging, regional lymph node (N) staging, distant metastasis (M) staging, ATA risk status, radioactive iodine (RAI) therapy, lymphatic invasion, and response to therapy at 1 year post initial treatment. Genetic alterations were categorized by driver. ATA, American Thyroid Association; CSTP, Comprehensive Solid Tumor Panel; PTC, Papillary Thyroid Carcinoma; TBSRTC, The Bethesda System for Reporting Thyroid Cytopathology.

Clinicopathologic features of the 147 patients with somatic oncogenic alterations are presented in Table 2 and Fig. 1. Patients with low-invasive alterations were less likely to demonstrate lymphadenopathy on preoperative ultrasound imaging (1/19, 5%) compared to patients with high-invasive alterations (70/128, 55%; *P* < 0.001). Similarly, tumors with low-invasive alterations were less likely to present malignant preoperative cytology (TBSRTC VI; 2/18, 11%) than those with high-invasive alterations (97/124, 78%; *P* < 0.001). The majority of patients (15/18, 83%) with low-invasive alterations who underwent FNA demonstrated indeterminate cytology (TBSRTC III–V). Of the nine lesions with high-invasive alterations having Bethesda III and IV cytology, 56% (5/9) had LN metastasis. Approximately 37% (7/19) of patients with low-invasive alterations underwent lobectomy or two-stage thyroidectomy, while 95% (121/128) of patients with high-invasive alterations underwent total thyroidectomy (Table 2).

**Table 2:** Clinicopathologic characteristics of pediatric patients with papillary thyroid carcinoma stratified by risk of driver alteration. Data are presented as *n* (%).

	Total, *n* = 147	Driver alteration		*P*
		Low-invasive, *n* = 19	High-invasive, *n* = 128	
Preoperative lymphadenopathy**^a^**				
Present	71 (48.3)	1 (5.3)	70 (54.7)	<0.001
Absent	71 (48.3)	17 (89.5)	54 (42.2)	
Unknown/not reported	5 (3.4)	1 (5.3)	4 (3.1)	
FNA performed^b^				
Benign (II)	3 (2.0)	1 (5.3)	2 (1.6)	<0.001
AUS (III)	6 (4.1)	2 (10.5)	4 (3.1)	
Follicular neoplasm (IV)	12 (8.2)	7 (36.8)	5 (3.9)	
Suspicious for malignancy (V)	22 (15.0)	6 (4.1)	16 (12.5)	
Malignant (VI)	99 (67.3)	2 (10.5)	97 (75.8)	
Not performed	4 (2.7)	1 (5.3)	3 (2.3)	
Unknown/not reported	1 (0.7)	0 (0.0)	1 (0.8)	
Age at time of thyroid surgery, years^c^	14.7 (12.9–16.8)	14.6 (14.2–15.8)	14.7 (12.9–16.9)	0.968
Surgery type				
Total thyroidectomy	133 (90.5)	12 (63.2)	121 (94.5)	<0.001
Lobectomy/isthmectomy	14 (9.5)	7 (36.8)	7 (5.5)	
Completion	8 (5.4)	3 (15.8)	5 (3.9)	
PTC histologic subtype				
Classic PTC	83 (56.5)	3 (15.8)	80 (62.5)	<0.001
Diffuse sclerosing variant PTC	16 (10.9)	0 (0.0)	16 (12.5)	
Follicular variant PTC	24 (16.3)	10 (52.6)	14 (10.9)	
Mixed PTC	12 (8.2)	4 (21.1)	8 (6.3)	
Oncocytic PTC	2 (1.4)	1 (5.3)	1 (0.8)	
Solid variant PTC	3 (2.0)	0 (0.0)	3 (2.3)	
Tall cell variant PTC	2 (1.4)	0 (0.0)	2 (1.6)	
Warthin-like PTC	1 (0.7)	0 (0.0)	1 (0.8)	
Not specified	4 (2.7)	1 (5.3)	3 (2.3)	

^a^Preoperative lymphadenopathy was determined through a review of thyroid ultrasound images and/or physical examination findings; ^b^FNA was classified according to the Bethesda System for Reporting Thyroid Cytopathology (TBSRTC). The highest category on primary tumor and/or lymph node(s) is reported. The proportion of malignant cytology was compared with two-tailed Fisher exact test; ^c^Values are median (IQR).

AUS, atypia of undetermined significance; FNA, fine needle aspiration; IQR, interquartile range (25th–75th percentile); PTC, papillary thyroid carcinoma.

Tumors with low-invasive alterations were more likely to be associated with low-invasive pathologic features and PTC subtypes associated with a lower risk for LN metastasis (34, 35). Of the patients with low-invasive alterations, 47% (9/19) were diagnosed with encapsulated-follicular variant PTC (enc-fvPTC). More than half of the patients (80/128, 63%) harboring high-invasive alterations demonstrated classic variant PTC (cPTC). Tumors harboring low-invasive alterations were significantly less likely to demonstrate multifocal disease (*P* = 0.008), bilateral disease (*P* = 0.010), extrathyroidal extension (*P* = 0.020), lymphatic invasion (*P* < 0.001), and extranodal extension (*P* < 0.001) compared to tumors with high-invasive alterations. No significant difference in vascular invasion (*P* = 0.916) was identified between the two cohorts (Table 3).

**Table 3:** Postoperative characteristics of pediatric patients with papillary thyroid carcinoma stratified by risk of driver alteration. Data are presented as *n* (%).

	Total, *n* = 147	Driver alteration		*P*
		Low-invasive, *n* = 19	High-invasive, *n* = 128	
AJCC TNM classification**^a^**				
Primary tumor (T)				
T1	63 (42.9)	6 (31.6)	57 (44.5)	0.147
T1a	22 (15.0)	3 (15.8)	19 (14.8)	
T1b	41 (27.9)	3 (15.8)	38 (29.7)	
T2	36 (24.5)	9 (47.4)	27 (21.1)	
T3	35 (23.8)	4 (21.1)	31 (24.2)	
T3a	21 (14.3)	4 (21.1)	17 (13.3)	
T3b	14 (9.5)	0 (0.0)	14 (10.9)	
T4a	12 (8.2)	0 (0.0)	12 (9.4)	
TX	1 (0.7)	0 (0.0)	1 (0.8)	
Regional lymph nodes (N)				
N0	38 (25.9)	17 (89.5)	21 (16.4)	<0.001
N0a	30 (20.4)	10 (52.6)	20 (15.6)	
N0b	8 (5.4)	7 (36.8)	1 (0.8)	
N1	109 (74.1)	2 (10.5)	107 (83.6)	
N1a	43 (29.3)	1 (5.3)	42 (32.8)	
N1b	66 (44.9)	1 (5.3)	65 (50.8)	
Distant metastasis (M)				
M0	113 (76.9)	17 (89.5)	96 (75.0)	0.140
M1	30 (20.4)	1 (5.3)	29 (22.7)	
Lungs	27 (18.4)	1 (5.3)	26 (20.3)	
Bone	2 (1.4)	0 (0.0)	2 (1.6)	
Brain	1 (0.7)	0 (0.0)	1 (0.8)	
Chest	1 (0.7)	0 (0.0)	1 (0.8)	
MX	4 (2.7)	1 (5.3)	3 (2.3)	
Tumor characteristics				
Focality				
Unifocal	63 (42.9)	14 (73.7)	49 (38.3)	0.008
Multifocal	83 (56.5)	5 (26.3)	78 (60.9)	
Unknown/not reported	1 (0.7)	0 (0.0)	1 (0.8)	
Laterality				
Unilateral	83 (56.5)	15 (78.9)	68 (53.1)	0.010
Bilateral	63 (42.9)	4 (21.1)	59 (46.1)	
Unknown/not reported	1 (0.7)	0 (0.0)	1 (0.8)	
Extrathyroidal extension				
Present	55 (37.4)	2 (10.5)	53 (41.4)	0.020
Microscopic	28 (19.0)	2 (10.5)	26 (20.3)	
Gross	27 (18.4)	0 (0.0)	27 (21.1)	
Absent	85 (57.8)	16 (84.2)	69 (53.9)	
Unknown/not reported	7 (4.8)	1 (5.3)	6 (4.7)	
Vascular invasion				
Present	58 (39.5)	7 (36.8)	51 (39.8)	0.916
Absent	83 (56.5)	11 (57.9)	72 (56.3)	
Unknown/not reported	6 (4.1)	1 (5.3)	5 (3.9)	
Lymphatic invasion				
Present	106 (72.1)	3 (15.8)	103 (80.5)	<0.001
Absent	35 (23.8)	15 (78.9)	20 (15.6)	
Unknown/not reported	6 (4.1)	1 (5.3)	5 (3.9)	
Extranodal extension				
Present	55 (37.4)	0 (0.0)	55 (43.0)	<0.001
Absent	77 (52.4)	18 (94.7)	59 (46.1)	
Unknown/not reported	15 (10.2)	1 (5.3)	14 (10.9)	
^131^I RAIT				
Received ^131^I RAIT				
Yes	114 (77.6)	8 (42.1)	106 (82.8)	<0.001
No	32 (21.8)	11 (57.9)	21 (16.4)	
Unknown/not reported	1 (0.7)	0 (0.0)	1 (0.8)	
Cumulative RAIT dosage, mCi^b^	103 (77–144)	104 (75–125)	103 (78–148)	0.968
ATA cancer risk stratification^c^				
Low risk	52 (35.4)	16 (84.2)	36 (28.1)	<0.001
Intermediate risk	41 (27.9)	2 (10.5)	39 (30.5)	
High risk	54 (36.7)	1 (5.3)	53 (41.4)	

^a^TNM was adopted from the AJCC 8 Edition Staging and evaluated within 12 months post-initial surgery; ^b^Values are median (IQR); ^c^Thyroid cancer risk stratification was adopted from the ‘2015 ATA Management Guidelines for Children with Thyroid Nodules and Differentiated Thyroid Cancer’ and evaluated at initial surgery. Extensive involvement was defined as >5 positive lymph nodes.

ATA, American Thyroid Association; AJCC, American Joint Committee on Cancer; IQR, interquartile range (25th–75th percentile); RAIT, radioactive iodine therapy; TNM, tumor–node–metastasis.

### Cervical lymph node involvement and distant metastasis

Patients with low-invasive alterations were less likely to have LNs removed from the central and/or lateral neck compared to patients with high-invasive alterations. Twelve patients (12/19, 63%) with low-invasive alterations had LNs dissected from the central neck compartment compared to 127 patients (127/128, 99%) with high-invasive alterations (*P* < 0.001). Of the low-invasive cohort of patients, 58% (11/19) underwent pCND, for which 91% (10/11) demonstrated no evidence of regional LN metastasis (AJCC N0a disease). One patient (1/19; 5%) had a therapeutic CND, and seven patients (7/19; 37%) did not undergo CND. Of the high-invasive cohort of patients, 36% (46/128) underwent a pCND, 60% (77/128) had therapeutic CND, and 4% (5/128) did not have a CND.

Patients with high-invasive alterations had a greater number of central neck LNs dissected than patients with low-invasive alterations, reporting a median of 14 LNs (IQR = 8–20) removed from patients with high-invasive alterations and a median of 6 LNs (IQR = 3–8) removed from patients with low-invasive alterations (*P* = 0.001; Table 4). Comparably, one patient (1/19; 5%) with a low-invasive alteration had LNs dissected from the lateral neck compartment compared to 73 patients (73/128, 57%) with high-invasive alterations (*P* < 0.001).

**Table 4:** Lymph node involvement in pediatric patients with papillary thyroid carcinoma stratified by risk of diver alteration. Data are presented as *n* (%) or as median (IQR).

	Total, *n* = 147	Driver alteration		*P*
		Low-invasive^a^, *n* = 19	High-invasive, *n* = 128	
CND				
Prophylactic	57 (38.8)	11 (57.9)	46 (35.9)	<0.001
Therapeutic	78 (53.1)	1 (5.3)	77 (60.1)	
Not performed	12 (8.2)	7 (36.8)	5 (3.9)	
Location of LN removal^b^				
Central compartment (levels VI–VII)				
Yes	139 (9.5)	12 (63.2)	127 (99.2)	<0.001
No	8 (5.4)	7 (36.8)	1 (0.8)	
Lateral compartment (levels II–V)				
Yes	74 (50.3)	1 (5.3)	73 (57.0)	<0.001
No	73 (49.7)	18 (94.7)	55 (43.0)	
Number of LNs removed^b^				
Total (levels II–VII)				
Positive	8 (1–20)	0 (0–0)	9 (2–22)	<0.001
Total	24 (11–49)	7 (3–9)	25 (13–52)	<0.001
Positive/total ratio	0.34	0.00	0.34	<0.001
Central compartment (levels VI–VII)				
Positive	6 (1–11)	0 (0–0)	6 (1–12)	<0.001
Total	13 (8–20)	6 (3–8)	14 (8–20)	0.001
Positive/total ratio	0.42	0.00	0.43	<0.001
Lateral compartment (levels II–V)				
Positive	7 (3–13)	1 (1–1)	7 (4–13)	<0.001
Total	31 (16–47)	16 (16–16)	32 (17–47)	<0.001
Positive/total ratio	0.23	0.06	0.22	<0.001

IQR, interquartile range (25th–75th percentile); LN, lymph node, pCND, prophylactic central neck dissection.

^a^One patient with a low-invasive *PAX8–PPARγ* fusion underwent a CND and selective removal of two LNs. Operative and pathology records report no metastatic LNs, however the total number of LNs removed was not reported and hence excluded from the quantitative analysis.

^b^pCND was indicated for patients having (i) no lymphadenopathy on preoperative ultrasound and/or physical examination by provider and/or (ii) FNA confirmation of benign LNs. Therapeutic LN dissection was indicated for patients having (i) central neck lymphadenopathy on preoperative ultrasound with FNA confirmation of malignancy or (ii) lateral neck lymphadenopathy on preoperative ultrasound with FNA confirmation of malignancy or (iii) preoperative ultrasound demonstrating rounded, echogenic LNs with peripheral blood flow along with an invasive-appearing lesion inside the thyroid with FNA confirmation of being suspicious for malignancy or malignant.

IQR, interquartile range (25th–75th percentile); LN, lymph node, pCND, prophylactic central neck dissection.

Overall, 84% (107/128) of patients with high-invasive alterations demonstrated metastatic LNs (N1a/N1b), compared to 11% (2/19) of patients with low-invasive alterations (*P* < 0.001; Table 3). Of the 42 patients (42/128; 33%) with N1a disease in the high-invasive cohort, 29 (29/42; 69%) did not have pre-operative lymphadenopathy identified on ultrasound and/or physical exam. Only two patients with low-invasive alterations (*BRAF^T599del^; TSHR^M453T^*) had positive metastatic LNs: 8/11 LNs+ (N1a) and 3/19 LN+ (N1b), respectively. Lymphadenopathy was identified preoperatively and confirmed on surgical pathology for the patient with* TSHR^M453T^*. Evaluation of LNs was limited preoperatively for the patient with* BRAF^T599del^*, the only patient in the low-invasive cohort found to have N1a disease despite negative preoperative US.

Twenty-nine patients (29/128; 23%) with high-invasive alterations demonstrated distant metastasis. Sites of distant metastasis in the high-invasive cohort included the lungs (26/128, 20%), bones (2/128, 2%), brain (1/128, 1%), and/or mediastinum (1/128, 1%). These tumors harbored *RET* fusions (14/29; 48%), *NTRK* fusions (9/29; 31%), *ALK* fusions (3/29; 10%),* BRAF^V600E^* (2/29; 7%), or *MET* fusion (1/29, 3%). By contrast, mild diffuse activity in the lungs was identified on a post-radiotherapy scan for one patient with a low-invasive *NRAS^Q61R^* variant. Pathological examination revealed an encapsulated, multifocal, and bilateral fvPTC with lymphatic and vascular invasion, which was subsequently staged as T2N0bM1. This patient did not have tissue available to assess for a combined *RAS-EIF1AX* variant, which may be associated with more invasive disease in adult patients (36).

Based upon the ATA pediatric PTC risk stratification, 84% (16/19) of patients with low-invasive alterations demonstrated ATA low-risk thyroid cancer, compared to 28% (36/128) of patients with high-invasive alterations (*P* < 0.001). Fifty-three patients (53/128; 41%) harboring high-invasive alterations demonstrated PTC classified as ATA high-risk.

## Discussion

We evaluated LN involvement in 147 pediatric PTC patients who demonstrated somatic oncogenic alterations over a 19-year study period. Our data support previous studies reporting that invasive tumor behavior, particularly the metastatic potential to regional LNs, correlates with somatic oncogenic alterations (19, 22). In the current study, only 11% (2/19) of PTC patients with low-invasive somatic oncogenic alterations demonstrated N1 disease, compared to 84% (107/128) of patients with high-invasive oncogenic alterations (*P* < 0.001; Table 3). Overall, 84% (16/19) of PTC patients harboring low-invasive alterations were categorized as ATA low-risk for persistent/recurrent disease, while 72% (92/128) of patients with high-invasive alterations (*BRAF^V600E^* and *RET, NTRK, ALK, BRAF,* or *MET* fusions) were categorized as ATA intermediate- or high-risk for persistent/recurrent disease (Table 3).

Forty-six patients (46/128; 36%) had a pCND in the high-invasive cohort. Of these patients, 61% (28/46) had LNM and no pre-operative evidence of central neck LN disease on pre-operative US, demonstrating oncogene analysis adds information about the metastatic potential that US and physical examination may miss. Furthermore, in nine patients with indeterminate cytology (Bethesda III or IV) and detection of a high-invasive oncogene, five patients (5/9, 56%) were found to have LNM – 60% (3/5) with N1a disease found on pCND and 40% (2/5) with N1b disease. Thus, pre-operative identification of an oncogene may be used to stratify which patients with indeterminate cytology may benefit from pCND. Of the 11 patients with pCND in the low-invasive cohort, 91% (10/11) were found to have no LN metastasis (N0a disease), showing there is potential to stratify patients out of the pCND category using somatic oncogene analysis.

Our observations are aligned with previous pediatric (17, 37, 38) and adult (39, 40, 41) studies evaluating the applicability of molecular oncogenic alteration classification to predict invasive behavior of PTC. While the Thyroid Cancer Genome Atlas (21) and other reports (39) have grouped PTC into *RAS*-like and *BRAF*-like oncogenic driver groups for low- and high-invasive behavior in adult PTC, pediatric-specific somatic oncogene analysis suggests that a three-tiered system more accurately describes increasing risk of invasive behavior: (i)* RAS*-like oncogenes (*PTEN*,* DICER1*,* RAS*,and *PAX8–PPAR*γ) are associated with a low risk for LNM; (ii) *BRAF^V600E^* is associated with a high risk for regional (N1) LNM; and (iii) fusion oncogenes (*RET*, *NTRK*, *ALK*, *BRAF*, or *MET* fusions) are associated with a high risk for regional (N1) and distant (M1) metastasis (17, 21, 39).

The results of our study hold potential clinical applicability as discussion remains over the benefit of pCND in the initial approach to management of pediatric patients with PTC. The 2015 ATA Pediatric Guidelines (5), as well as several recent publications (42, 43), rely on N status to stratify the extent of initial surgery (43) and RAIT (5, 42). There are, however, concerns over increased surgical complications associated with pCND (12), with the recent European Thyroid Association Guidelines for the management of pediatric thyroid nodules and differentiated thyroid carcinoma recommending to limit pCND to patients with ‘suspicious features of advanced thyroid cancer’ (13). Preoperative cytopathology may aid in patient selection, limiting pCND to patients with TBSRTC V and VI cytology (20). However, the invasive behavior of PTC, including sonographic assessment of LN disease and cytological classification, is subjective with wide variation across institutions limiting the ability to make generalized recommendations based solely on US and cytology (15). The incorporation of somatic oncogene testing into the preoperative assessment may afford an opportunity to limit pCND for patients with nodules harboring low-invasive somatic alterations that demonstrate low-risk features on preoperative ultrasound (i.e. smooth margins and wider-than-tall shape and no evidence of extrathyroidal extension, punctate echogenic foci, or regional adenopathy (N0b status)) and TBSRTC indeterminate cytology (categories III/atypia of undetermined significance and IV/follicular neoplasm) (20).

The limitations of this study are its single-center retrospective design, variance in somatic oncogenic panels, a lower percentage of patients with low-invasive alterations undergoing CND compared to patients with high-invasive alterations, and lack of somatic oncogene data for all tumors. Despite these limitations, the data on the lower rate of central neck LNM in tumors with low-invasive somatic oncogenic alterations are intriguing (Table 4) and support the potential benefit of prospective studies exploring the utility of preoperative molecular testing to stratify surgical approach to care.

Current efforts are underway to increase the sample size and perform multi-omics analysis to further delineate the genomic landscape of pediatric PTC beyond somatic oncogene driver. One of the goals of this initiative is to identify additional markers of invasive behavior for the 16% (21/128) of tumors that were found to have a high-invasive somatic alteration but did not have metastasis to the central neck compartment (N0; Table 3). Prospective, multi-center studies are warranted to confirm whether the incorporation of all available data, including ultrasonography, cytology, and somatic oncogene, can be incorporated into clinical practice in an effort to reduce potential surgical complications of pCND without compromising the ability to stratify RAIT based on current pediatric risk stratification systems or to achieve remission from disease (5, 42). To this end, the authors have helped create the Child and Adolescent Thyroid Consortium (CATC), an international pediatric thyroid consortium designed to enhance collaboration between multidisciplinary pediatric thyroid centers (www.thyroidcatc.org).

## Conclusion

Pediatric patients with low-invasive somatic oncogenic alterations are at low-risk for metastasis to central neck LNs. Our findings suggest that preoperative knowledge of somatic oncogene alterations provides objective data to stratify pediatric patients who may not benefit from pCND. Future prospective studies are needed to validate if comprehensive somatic NGS panels for pediatric patients with indeterminate cytology can be used to optimize surgical management, limiting pCND to tumors with high-invasive somatic variants and fusions and not performing pCND for tumors with low-invasive alterations.

## Declaration of interest

The authors declare that there is no conflict of interest that could be perceived as prejudicing the impartiality of the study reported.

## Funding

This work was supported in part by The Children’s Hospital of Philadelphia Frontier Program (grant no. 000000495).

## Ethics

This retrospective study involving human subjects was reviewed and approved by the Children’s Hospital of Philadelphia Institutional Review Board (CHOP IRB #17-014224). Written informed consent from the participant and/or participant’s legal guardian was not required per CHOP IRB; a waiver of consent/parental permission has been approved per 45 CRF 46.116(d).

## Author contribution statement

AJB devised the project. JAB, MB, AI, and SH collected the data and performed the analysis. JAB, JCRF, and SH created the tables and figures. JAB and MB wrote the manuscript, with support and critical review provided by SH, AJB, JCRF, AI, MB, AF, KK, NSA, and SMM. All authors discussed the results and provided editorial review of the manuscript.
